# Whole blood pathogen reduction technology and blood safety in sub-Saharan Africa: A systematic review with regional discussion

**DOI:** 10.4102/ajlm.v5i1.363

**Published:** 2016-06-09

**Authors:** Asa’ah Nkohkwo, Gabriel Agbor, Emmanuel Asongalem, Claude Tagny, Tazoacha Asonganyi

**Affiliations:** 1 N-S TechnoMed, London, United Kingdom; 2 Department of Biochemistry, Institute of Medical Research and Medicinal Plants Studies, Yaoundé, Cameroon; 3 Department of Biomedical Sciences, Faculty of Health Sciences, University of Buea and Toxicology Society, Buea, Cameroon; 4 Haematology and Blood Transfusion Service, University Teaching Hospital, Yaoundé, Cameroon; 5 Department of Biochemistry and Immunology, Faculty of Medicine and Biomedical Sciences, University of Yaoundé 1, Yaoundé, Cameroon

## Abstract

**Background:**

Despite vast improvements in transfusion services in sub-Saharan Africa over the last decade, there remain serious concerns on the safety and adequacy of the blood supply across the region.

**Objective:**

This review paper ascertains the role of pathogen reduction technology (PRT) in improving blood safety and supply adequacy in the region.

**Method:**

The state of blood safety in sub-Saharan Africa was reviewed. Meetings, seminars and correspondence were undertaken with key clinicians, scientists and professional bodies in the region, including the World Health Organization’s Regional Office for Africa, to examine the suitability of PRT for improving the safety of whole blood transfusion, a prevalent transfusion format in the region.

**Results:**

Existing literature suggests that combining PRT with current blood safety measures (such as serology) would improve the safety and adequacy of the blood supply for transfusions in sub-Saharan Africa. This was echoed by the findings of the stakeholder meetings.

**Conclusion:**

Following a detailed appraisal of two leading PRT systems, the Mirasol^®^ PRT System and the Cerus S-303 System, we suggest that companies conduct comprehensive toxicological evaluation of the agents used for PRT and publish this in the scientific literature. We also recommend that the safety and efficacy of these technologies should be established in a randomised clinical trial conducted in sub-Saharan Africa.

## Introduction

The United Nations Millennium Declaration, issued in 2000, led to the establishment of eight international development goals, which became known as the Millennium Development Goals.^1^ The intention of the 193 member states of the UN was to meet these goals by 2015 by: (1) reducing child mortality rates; (2) improving maternal health; and (3) combatting HIV, malaria and other diseases.^1^ Establishing an adequate supply of safe blood for transfusion would improve the likelihood of achieving these three goals, especially in low- and middle-income countries, many of which are situated in sub-Saharan Africa.^2^

The World Health Organization (WHO) estimates that there are 3.9 whole blood donations per 1000 inhabitants in low-income countries; far below the minimum WHO requirement of 10 whole blood donations per 1000 inhabitants.^2^ The WHO has estimated that in low-income countries the prevalence of infectious diseases such as HIV is 425 times greater than in high-income countries.^3,4,5^ Testing methodologies also vary significantly between these regions. In high-income countries, almost all blood samples are tested with the highly-sensitive nucleic acid testing (NAT),^3,4,5^ whereas low- and middle-income countries still rely on various serological methods and rapid tests, which are less sensitive and thus have a greater window period than NAT screening. Moreover, most laboratories in low-income countries do not perform tests in a quality-assured manner.^3,4,5^ Indeed, this has necessitated accreditation and mentoring initiatives, such as the establishment of the African Society for Laboratory Medicine in 2011. Thus, patients in low- and middle-income countries are at higher risk for transmission of infection through blood transfusion than patients in high-income countries.^3,4,5^ Whilst improvements have been made in the past, a high residual risk of transfusion-transmitted infections (TTIs) remains because of viruses, bacteria, protozoa and residual contaminating leukocytes, despite decades of blood safety programmes.^3,4,5,6,7,8^

Finally, whereas in high-income countries almost all blood is fractionated into plasma, platelets and red blood cells (RBCs),^9,10^ in sub-Saharan Africa, approximately two million of the three million units per year are still transfused as whole blood.^9,10^ Where they are componentised, the primary requirement is for RBCs, followed by plasma.^9,10^ In 2010, only 31.5% of blood centres in the WHO’s African Region prepared blood components.^9,10^

### Blood safety in sub-Saharan Africa

In recent years, there has been a vast improvement in the organisation, management, clinical and technical aspects of transfusion services in sub-Saharan Africa,^9,10,11^ although serious concerns remain with regard to the safety and adequacy of the blood supply.

According to the WHO recommended minimum of 10 units of donated blood per 1000 catchment population, an estimated 8–9 million units of blood are currently needed per year for transfusion in Africa.^11^ In sub-Saharan Africa, 44% of maternal deaths are attributed to severe bleeding during pregnancy and childbirth.^11^ Thus, the majority of blood products are used for treating pregnancy-related bleeding.^11^ Another major group that receives blood transfusions are children; 50% of paediatric transfusions are for malaria-induced anaemia in children and other severe morbidities, notably anaemia from sickle-cell disease.^12^

Improving access to safe blood leads to improved healthcare. In Malawi, safer blood led to a 60% decrease in mortality amongst seriously ill children and a 50% decrease in mortality amongst pregnant women with severe blood loss.^13^ Many factors in sub-Saharan Africa contribute to the low availability of safe blood. Two major factors are low donation rates and a high prevalence of TTIs.^2,3,4,5,8,9^

The blood donation rate in sub-Saharan Africa is generally low, with 4–5 per 1000 population^8,9^ compared with 30 donations per 1000 in developed countries.^2^ Thus, only 40% of targeted blood supply needs were realised by collection services in 2010.^2,9,14^ According to WHO data,^8,9^ blood transfusion from regular voluntary non-remunerated blood donors (VNRBDs) have the lowest risk of TTIs. However, VNRBDs represent less than 50% of whole blood donations in low-income countries compared with 76% – 100% in high-income countries.^8,9^ The low VNRBD levels in some African countries may be a result not only of lack of organisation and financial resources, but also, to some extent, of socio-cultural barriers such as limited levels of education, religious and mystic beliefs and misconceptions about blood use.^15^

The high prevalence of TTIs, such as infection with HIV (5% amongst adults), hepatitis B virus (8%) and hepatitis C virus (10%),^9^ contributes to the poor supply, in terms of adequacy and safety.^2,3,4,9,10^ Also important to note is that, of the estimated one million annual deaths resulting from malaria globally, 90% occur in sub-Saharan Africa.^7,9^ This suggests a high carrier rate of malarial parasite in this region and hence in the pool of potential blood donors. According to the respondents of a 2010 survey of the status of blood safety in the WHO African Region, an estimated 7.5% of blood units were discarded to avoid the risks of TTIs.^9^ In high-income countries, donor screening and deferral procedures, as well as serologic testing and NAT, have helped to make blood a safer product, drastically reducing the risk of classical TTI agents such as HIV and hepatitis viruses.^3,4^ However, whilst highly-sensitive techniques such NAT are available in high-income countries, screening and testing in low- and middle-income countries where the risk is highest, are limited to a combination of donor screening and/or deferral and serological procedures. High residual risks have been reported in African blood banks where NAT is not performed.^4^ NAT would allow identification of contaminated blood from donors who are in pre-serological phases of disease. However, this additional screening test is usually too expensive for the limited budgets of African blood services.

Another major difference between blood transfusion in low- and middle-income countries and high-income countries is that only 41.2% of all collections in Africa (1.4 million of 3.4 million) were transfused as components in 2010.^9^ Although there is increased use of components in sub-Saharan Africa, with the greatest need being for RBCs, it is expected that whole blood transfusions and red cell concentrates will continue to be the most requested units in the near future.^10,12^

### Aim

The aim of this review was to determine the suitability of pathogen reduction technology (PRT) for improving the safety of whole blood transfusions in sub-Saharan Africa, as well as for improving the adequacy of the blood supply available for transfusions. This technology could offer a way forward toward addressing the Millennium Development Goals in sub-Saharan Africa through helping to ensure an adequate and safe blood supply.

## Review method

During the period September 2012 to January 2015, we searched for articles in the PubMed index using keywords and combinations of keywords, including ‘pathogen reduction/inactivation’, ‘blood safety’, ‘transfusion-transmitted infections’, ‘sub-Saharan Africa’, ‘Mirasol’, ‘S303’, ‘riboflavin’ and ‘amustaline’. We also sought out articles from emerging key opinion leaders. We then applied the Grading of Recommendations, Assessment, Development and Evaluation (GRADE)^16^ guidelines to undertake a qualified review of the collected publications^17^ on the challenges of blood safety in sub-Saharan Africa. We considered the potential of nascent PRT as a possible solution for the challenges.^18,19,20^ The two most promising novel interventions, the Mirasol^®^ PRT and the Cerus S-303 PRT, were examined, as these two systems could offer additional solutions to ensure safer transfusion of whole blood, potentially leading to a paradigm shift for healthcare in low- and middle-income countries.^18,19,20^ The use of GRADE enabled us to avoid bias and be as objective as possible in considering the information and literature we assessed. The dearth of randomised controlled trials, as recommended by GRADE, meant that the literature findings had to be verified. Hence, to triangulate our observations, and given the target consumer region of the two leading PRTs, we engaged key stakeholders in the region. We sought expert opinions by discussing the findings of our literature search with clinicians, scientists and various professional bodies, including the WHO’s Regional Office for Africa, at seminars in Brazzaville, Republic of Congo and Yaoundé, Cameroon.^21,22,23^

## Findings

### The challenges of an inadequate and unsafe supply of blood for transfusions

The situation described in the WHO 2010 survey^9^ was reinforced by the statements and presentations at the meetings in Brazzaville and Yaoundé^21,22,23^ and is typified by the current state of affairs in Cameroon,^24^ Ghana,^5^ Nigeria^7^ and the rest of sub-Saharan Africa.^5,15^ Two key challenges in the region were highlighted: the overall inadequacy of the blood supply and the lack of safe blood for transfusions. These challenges are faced not only by women with pregnancy-related problems and children with paediatric malaria-induced anaemia,^8,9^ but also by individuals with other severe, debilitating conditions such as sickle-cell disease. Sickle-cell disease is a genetic haemoglobinopathy, manifesting typically as life-threatening episodes or ‘crises’ (including a combination of moderate to severe pain, anaemia and other acute complications) related to venous occlusion.^25,26^ With an estimated 200 000 infants born every year in Africa with sickle-cell disease and an estimated prevalence of 2%,^27^ there are approximately 20 million people living with sickle-cell disease in sub-Saharan Africa. Because of the region’s growing population, this number will continue to increase. The effective management of sickle-cell disease in other regions suggests that 10% of a given population of sickle-cell disease patients require a life-saving transfusion each year to prevent strokes in children or to address acute complications in other groups.^27,28^ Prognosis is poor across sub-Saharan Africa, with an 80% five-year mortality rate amongst children with sickle-cell disease, resulting from the lack of resources to ensure appropriate and safe management of life-threatening anaemia, amongst other issues.^27,29^

Thus, added to the wider iatrogenic risks such as TTIs associated with transfusions,^5,6,7,8,9^ the shortage of blood denies sickle-cell disease and many other patients in sub-Saharan Africa the adequate healthcare that is taken virtually for granted in Europe and North America.

### The potential of pathogen reduction technology

There are a number of current technologies for the reduction of blood-borne pathogens.^19,20,21^ These include systems for platelet concentrates and plasma, such as the Cerus Intercept system, which is based on psoralen; the Theraflex system, which is based on UVC irradiation; the Mirasol system, which is based on riboflavin and ultraviolet light; and the Cerus S-303 system, which is based on a DNA crosslinker. Presently, only two of the most prominent PRT platforms have the potential for whole blood treatment: the Mirasol PRT system for Whole Blood^30^ and the Cerus S-303 system.^31^ A phase-III study has recently been completed for the Mirasol system for whole blood.^32,33^ Thus, given the prominence of the whole blood transfusion format in sub-Saharan Africa, only the Mirasol PRT and Cerus S-303 systems were considered potentially suitable at the Brazzaville and Yaoundé stakeholder meetings.^18,19,20^

## Discussion

### Ensuring blood safety in sub-Saharan Africa

Three key principles^18,19,20,34^ would protect recipients from donor blood pathogens: improved donor selection/exclusion measures; improved and standardised methodology of testing; and reduction/removal of pathogens from the blood.

With regard to donor selection and/or exclusion, it should be pointed out that the WHO promotes VNRBD.^35^ The WHO also sees the practice of family replacement donations, as exemplified in Cameroon,^24^ as a risk to the blood supply because of the perceived potential of paid blood donation. However, there is some disagreement on the risk of this practice within the context of low-income countries, as some view the primary problem more as lack of available blood.^36^ Family donation may not increase the risk to the blood supply in comparison to VNRBDs.^36^ Furthermore, the establishment of VNRBDs as the only source of blood, as a policy, may not only increase the cost of a unit of blood by two- to five-fold and exacerbate the pre-existing blood shortage, but would hardly meet local blood supply needs.^36^ It is also questionable whether hospital-based family replacement blood banks in Africa would ever be able to maintain adequate blood supplies to meet local needs. Some have reported that first-time VNRDs are no safer than family/replacement donors.^35^

As for the testing of donated blood, the current practice in high-income countries is to employ the following layers of protection: donor screening, serology, NAT, leukoreduction, gamma irradiation and bacterial detection. Donors in high-income countries have a much lower prevalence of TTIs.^37,38^ The outcome is that risk for TTIs in high-income countries is low for tested pathogens.^37,38^ Typically, residual risk ranged from 1 in 300 000 units for hepatitis B virus in the United States, to 1 in 8 million units for HIV in Canada, to 1 in 11 million units for hepatitis C virus in Germany.^37,38^ In contrast, in sub-Saharan Africa, residual risks were 4.3 in 1000 units for hepatitis B virus, 1 in 1000 units for HIV and 2.5 in 1000 units for hepatitis C virus from blood transfusion.^39^ The WHO further reported inadequate quality assurance of donated blood, including unreliable vetting of the donor and serological testing of the donated blood, coupled with the practice of whole blood transfusion.^13^

Considering all of these factors, the current status of highly risky transfusion in sub-Saharan Africa requires a new paradigm approach. To start a combination of new technologies is needed that would be effective for whole blood transfusion – a significant format of transfusion in sub-Saharan Africa – and that could enhance the current testing solution for improved safety. Given the difficulty of recruiting sufficient donations, the proposed way forward should also have the potential to reduce supply wastage by rendering safe blood that would otherwise be rejected for testing positive. Amongst other attributes, the appropriate technology would need to exhibit: safety, effectiveness, clinical efficacy, physiological integrity of biological components, operational efficiency and competitive health economics. The various platforms encompassing PRT represent such a novel approach.^18,19,20^ PRT platforms are already in use for platelets and plasma in many countries throughout Europe,^30,31^ Asia and the Americas. With the emergence of platforms suitable for whole blood use,^18,19,20,30,40^ PRT now has the potential to offer an additional step in the standard blood safety value chain in sub-Saharan Africa. Moreover, the ability to inactivate pathogens in whole blood offers the flexibility of either using the blood as whole or, after inactivation, to subsequently separate the blood into components for storage.^40^

### Moving forward: validation and safety of pathogen reduction technology

We examined two PRT platforms in detail, the Mirasol PRT System for Whole Blood and the Cerus S-303 system. Both systems are based on disrupting the nucleic acid in the pathogen. The Mirasol system achieves this through electron transfer reactions, whereas the Cerus S-303 system does so through irreversible crosslinking of the DNA. We focused specifically on the toxicology of the systems to laboratory staff and patients and the potential usefulness of these two systems for whole blood transfusion.

#### The Mirasol PRT System for Whole Blood^30,38^

**FIGURE 1:**
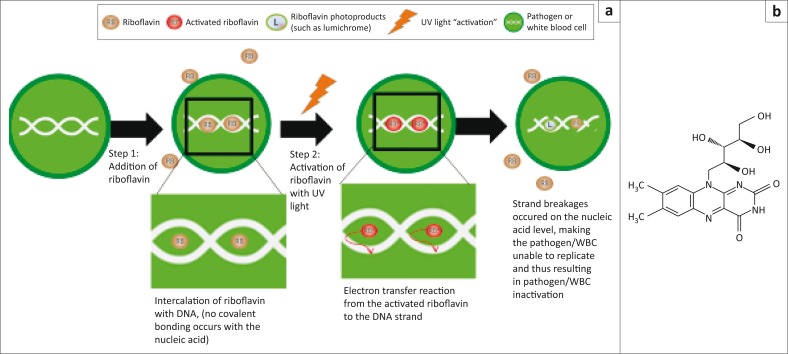
(a) Mode of action of the Mirasol PRT system; (b) Structure of riboflavin.

**The system:** The Mirasol PRT system is manufactured by Terumo BCT (Lakewood, Colorado, United States) and was granted the CE mark in 2008.^30^ The system employs riboflavin (vitamin B2) and ultraviolet illumination to inactivate a range of known and unknown pathogens.^41^ Riboflavin first binds with the nucleic acids in the pathogen, then the ultraviolet illumination of the vitamin B2–pathogen complex causes an irreversible chemical reduction (specifically, guanine oxidation), disrupting the DNA and consequently inactivating the pathogen (Figure 1).

**Safety:** Riboflavin is a safe, non-toxic, non-mutagenic, water-soluble vitamin necessary for normal cell function, growth and energy production in humans.^42^ Used in vital metabolic processes, it is found in most animal and plant tissues and is considered an essential part of the human diet.^42^ The Institute of Medicine of the United States National Academy of Sciences recommends a dietary intake for riboflavin of 1.1 to 1.3 milligrams per day for adults.^42^ Studies have shown that excess riboflavin is rapidly excreted in the urine; consequently, a minimal amount is stored in the body.^42^ Riboflavin deficiency may contribute to increased concentrations of plasma homocysteine, which is associated with an increased risk of cardiovascular disease.^42^ Amongst other morbidities, it may also be associated with impaired handling of iron and night blindness. Riboflavin and its photoproducts produced using the Mirasol system are present in untreated human blood.^43^ This has been demonstrated using high performance liquid chromatography where Mirasol-treated platelet concentrates and untreated platelet concentrates were compared.^43,44^ The analysis indicated that no new photoproducts were formed during the Mirasol system process.^43,44^

Extensive toxicology studies have been performed to confirm the safety profile of riboflavin.^42^ In addition, riboflavin photoproducts that are generated from the Mirasol process, such as lumichrome, can be transfused at several degrees of magnitude lower than the toxic concentration. Further, a large, well-controlled study of Mirasol-treated platelets, as well as a haemovigilance study on Mirasol-treated products, have shown no adverse events attributed to the use of Mirasol. Of importance is a study at the Institute of Health and Tropical Medicine in Warsaw, Poland,^43^ that followed six patients with thrombotic thrombocytopenic purpura who received a total of 711 Mirasol-treated fresh frozen plasma units. The massive transfusions of Mirasol-treated fresh frozen plasma were found to be safe and effective when used for therapeutic plasma exchange in the treatment of thrombotic thrombocytopenic purpura.^43^

The chemistry of the base technology for the Mirasol PRT platform remains the same for whole blood, plasma and platelets.^30,38^ Concerns have been expressed that photo-treatment is limited in its application to materials containing RBCs. The absorption of light by haemoglobin in several regions of the ultraviolet and visible spectra results in the photo-treatment of RBCs and alters the cells in a similar manner. However, most of the applied energy occurs in the UVB range (280–315 nm) with peak wavelength at 313 nm, which is different from the absorbance energy of mitochondrial enzymes (370–450 nm). Thus, the oxidative phosphorylation pathway is not affected.^45,46^ Indeed, photo-treatment does not have any effect on the quality of RBCs for transfusion.^47,48^

**Strengths and challenges:** The Mirasol platform has been licensed and is being used across Europe for plasma and platelet transfusions.^30^ It has also recently been tested in a phase III trial in Ghana for preventing malaria associated with whole blood transfusion.^32,33^ In Ghana, up to 28% of all blood is contaminated with *Plasmodium* spp. parasites.^5^ The phase III trial reported that Mirasol-treated whole blood showed a statistically- and clinically-significant reduction in transfusion-transmitted malaria compared with the control group that received untreated whole blood.^32,33^ In addition, the number of transfusion-related adverse events from the transfused blood products was similar between the treated and control groups.^32,33^ This clinical trial demonstrated the potential of PRT for sub-Saharan Africa, showing that even for untested pathogens, PRT can significantly reduce the incidence of TTIs.

Because the Mirasol PRT is a nascent technology, the blood-banking sector, long-accustomed to routine traditional testing, would need to take into consideration the substantial paradigm shift and the training of personnel toward ensuring its safe and effective implementation. In addition, the extra cost of combining the new technology with current routine testing remains a key limitation.

#### The Cerus S-303 blood system^49,50,51^

**FIGURE 2:**
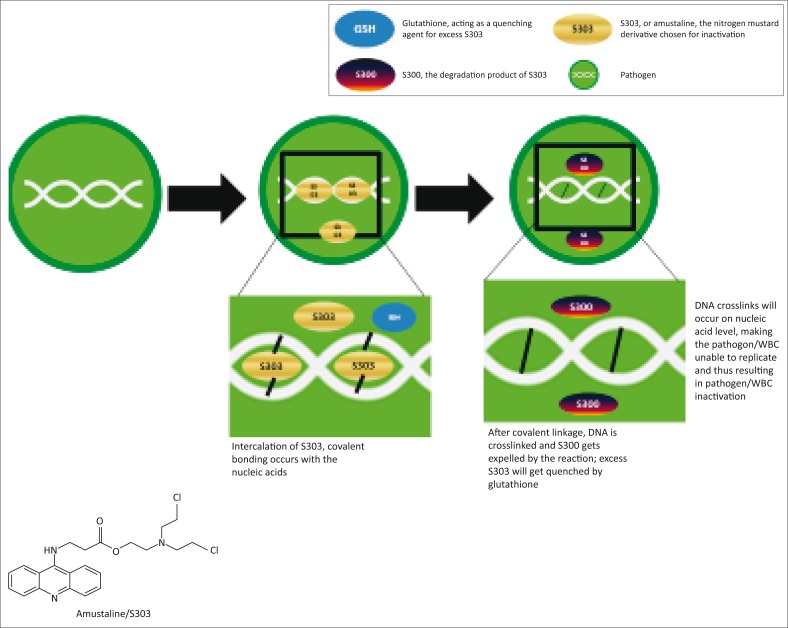
Mode of action of the Cerus S-303 system.

**The system:** The Cerus S-303 system is manufactured by Cerus Corporation (Concord, California, United States) and a CE mark was granted in 2002.^31^ The Cerus system (Figure 2) employs a chemical compound, S-303 (amustaline), which intercalates with the nucleic acids in the pathogen with no activation of the material.^49,50,51^ The system uses the S-303 frangible anchor linker effector (FRALE) method, which includes a nucleic acid binding component that serves as anchor, a nucleic acid reactive group (the effector) and a hydrolysable linker.^49,50,51^ The FRALE compound targets nucleic acids, whereas the effector moiety, a nitrogen mustard, results in crosslinking (through the effector) of the nucleic acid, thus preventing replication and inactivating the pathogen. After the reaction, the material decomposes through a pH-dependent process into an unreactive product, S-300.^51^ Glutathione is used to quench the side reactions of the effector with other biological materials.^49^

**FIGURE 3:**
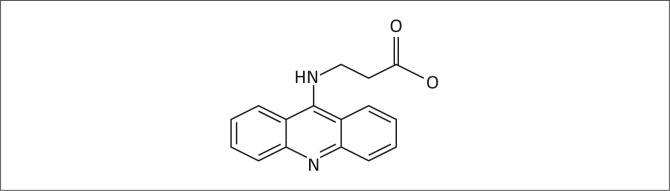
S-303 reaction product: S-300.

**FIGURE 4:**
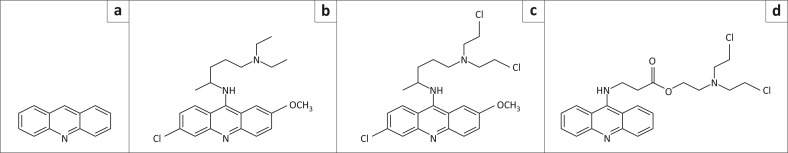
Members of the acridine family of compounds: (a) Structure of acridine; (b) Structure of acridine family member quinacrine; (c) Structure of acridine family member quinacrine mustard; (d) Structure of acridine family member S-303.

**Safety:** The Cerus platform uses a xenobiotic/foreign chemical, S-303, as the pathogen reduction agent, which in turn results in xenobiotic breakdown products, including S-300 and products of the ‘heteroalkyl’ group.^52^ Quinacrine and quinacrine mustard are structural analogues of S-303 (Figures 3 and 4). The pharmacokinetic properties of quinacrine are well known.^53^ Following administration, it is rapidly absorbed and distributed in the body. Plasma levels remain low compared with tissue concentrations,^54^ with high concentrations in the liver, spleen, lungs and adrenal glands. Liver concentrations can reach 20 000 times concentrations in plasma.^55,56,57^ The brain, heart and skeletal muscles have low concentrations of quinacrine;^55,56^ and significant deposits are found in the skin, fingernails and hair.^57^ Over 80% – 90% of quinacrine is bound to plasma proteins, whereas cerebrospinal fluid levels are 1% – 5% of plasma levels with a half-life of 5–14 hours, depending on dosage.^58^ Synthetic structural analogues of quinacrine, such as quinacrine mustard and S-303 and the latter’s metabolic product (S-300), would probably share some of these attributes.^59^ The S-300 study report^51^ did not measure the amount of S-300 deposited in various organs and tissue. Such short-term toxicological studies are not likely to reveal rare or slow effects, such as carcinogenicity, or other possible consequences of genotoxicity.

Furthermore, quinacrine, quinacrine mustard, S-303 and S-300 are heterocyclic amines, which are known for their mutagenicity.^60,61^ Quinacrine and quinacrine mustard interact with bovine heart mitochondrial F1-ATPase,^62^ with quinacrine mustard potently inactivating the enzyme.^63^ Further, quinacrine and quinacrine mustard bind to mouse muscle nicotinic receptors of acetylcholine, irreversibly inhibiting response,^63^ as well as to axonal membranes of nerve cells,^64^ interfering with the conduction of nerve impulses. Quinacrine mustard also has cytostatic and cytotoxic properties.^65^

It is well known that compounds with structures similar to a pharmacologically-active drug are often themselves biologically active.^59^ Therefore, we are concerned about the striking structural similarity of S-303 to substances like quinacrine mustard, quinacrine and acridine orange that are known to be either in the mustard group of compounds or in the heterocyclic amine group, which is also known for having cytotoxic and genotoxic properties.

Some patients transfused with S-303-treated RBCs developed positive cross-match reactions to the RBCs with the first generation of the Cerus platform. As a result, the second generation system includes glutathione as a quencher.^65^ As stated by the manufacturers, glutathione binds to S-300^65^, the reaction product, to prevent it from reacting with proteins. Glutathione does not enter RBCs, but remains in the external environment where it prevents S-300 from reacting with plasma proteins.^52^ However, *in vivo*, glutathione is found predominantly in RBCs, not in plasma.^51^ If whole blood treated with S-303 were to be transfused into patients, the glutathione in the treated sample would be diluted *in vivo* and no longer available to prevent S-300 from reacting with the vital biological components of the extracellular environment.

**Strengths and challenges:** The Cerus Intercept platform version of the Cerus S-303 is currently available for platelets and plasma. A phase III clinical trial for RBCs has been completed in Europe.^20,50^ Thus, similar to the Mirasol PRT system, the Cerus S-303 platform is potentially useful for whole blood applications, making it another strong contender for reducing TTIs in situations where whole blood transfusion is traditional, as is the case in sub-Saharan Africa.

That said, there is a need for more appropriate experimental approaches to determine adverse effects such as immunotoxicity, carcinogenicity and genotoxicity. Importantly, the current absence of a removal step in the whole blood PRT process proposed for packed RBC treatment for the S-303 platform only emphasises the need for such detailed toxicological evaluations. Indeed, further extensive toxicological evaluation of S-303 and its breakdown products, in phase III of the evaluation process, would ensure not only safety but also a reproducible and user-friendly technology for resource-limited settings. Such technology should be adapted, not only to current recommended blood safety regulations, but should include ethical considerations, affordability and economical sustainability for blood services.^66^ Thus, as in with the Mirasol system, addressing toxicity considerations in addition to the extra cost of combining the Cerus S-303 technology with current routine testing remain key limitations.

### Pathogen reduction technology and the pathogen detection window

**FIGURE 5:**
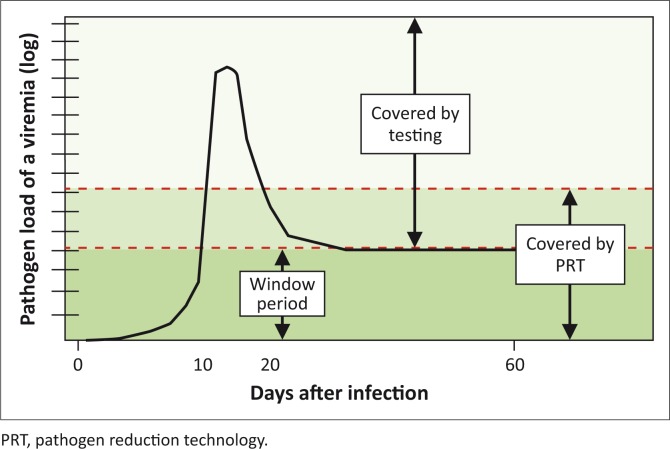
The principle of combining pathogen reduction technology with existing testing methods.

The above limitations of toxicity (Cerus S-303 platform) and extra cost (both the Mirasol and Cerus S-303 platforms) notwithstanding, based on the underpinning pathogen nucleic binding chemistry, both PRT formats are potentially effective against a range of pathogens known and unknown, on white blood cells, as long as they contain nucleic acids. Moreover, by virtue of the underlying analytical sensitivity of the strategy, PRT has the potential of further closing the pathogen detection window period – when blood levels of specific pathogen markers are still below the detection limits of other methods (Figure 5).

This highlights the potential of PRT to markedly improve the safety of blood through a synergistic combination with the diagnostic window of testing. This conclusion came out of the seminars held with stakeholders, including clinicians, scientists and the WHO’s Regional Office for Africa, during the period of this study.^21,22,23^

## Conclusion

### Whole blood transfusion and adequacy of the blood supply

Given the high cost of establishing and running a blood componentisation programme, it might be beneficial for low- and middle-income countries to keep their focus on whole blood transfusions, increase blood donations and decrease the risk of exposure to pathogens in the blood supply, rather than on extensive componentisation. However, facilities permitting, the need for blood components might be met through apheresis, allowing blood banks to produce specific blood components based on the demand of the prescribing physician. That said, reduction of blood transfusion to their indispensable indications and appropriate use of blood components are critical to meet safe blood needs. This includes early diagnosis of diseases requiring blood transfusion, the use of surgical techniques and of medication aimed at limiting blood loss, as well as respect for national directives. In 2009, less than one out of 10 African countries had a proper policy in place for clinical use of blood.^9^ Inappropriate use of blood leads to blood shortages and increased risk of TTIs.

### A new safety paradigm toward whole blood pathogen reduction technology

The provision of an adequate blood supply and safe transfusion of blood as recommended in the United Nations’ Millennium Development Goals remains a major challenge for sub-Saharan Africa. Current blood-borne pathogen testing arrangements, hampered by lack of adequate testing resources, result in TTIs at an estimated risk of 10% – 50% per transfusion. Moreover, because of poor blood donation rates, blood supplies at blood banks are low. This review supports the position that PRT may offer a solution for ensuring that the regionally dominant format of whole blood transfusion is rendered safer by removing TTIs. We suggest that a new paradigm toward combining PRT with standard quality testing would improve the safety of transfusion. The existing literature suggests that combining PRT with current routine blood screening methods (such as serology) would improve the safety of transfusions in sub-Saharan Africa. Following a detailed review of two of the leading PRT methods, the Terumo BCT Mirasol PRT Whole Blood system and the Cerus S-303 system, we suggest that manufacturers conduct a comprehensive toxicological evaluation of the agents used for PRT and publish their findings in the scientific literature. We also recommend that the safety and efficacy of PRT technology should be established in a randomised clinical trial conducted in sub-Saharan Africa, if the technology is adopted in the region.
